# Abnormal Complement Activation and Inflammation in the Pathogenesis of Retinopathy of Prematurity

**DOI:** 10.3389/fimmu.2017.01868

**Published:** 2017-12-22

**Authors:** Sonika Rathi, Subhadra Jalali, Satish Patnaik, Shahna Shahulhameed, Ganeswara R. Musada, Divya Balakrishnan, Padmaja K. Rani, Ramesh Kekunnaya, Preeti Patil Chhablani, Sarpras Swain, Lopamudra Giri, Subhabrata Chakrabarti, Inderjeet Kaur

**Affiliations:** ^1^Prof Brien Holden Eye Research Centre, Hyderabad, India; ^2^Smt. Kanuri Santhamma Centre for Vitreo Retinal Diseases, Hyderabad, India; ^3^Jasti V Ramanamma Children’s Eye Care Centre, L V Prasad Eye Institute, Hyderabad, India; ^4^Indian Institute of Technology, Hyderabad, India

**Keywords:** retina, premature birth, inflammation, genetics, cytokines, abnormal angiogenesis, microglia/macrophage, alternative complement pathway

## Abstract

Retinopathy of prematurity (ROP) is a neurovascular complication in preterm babies, leading to severe visual impairment, but the underlying mechanisms are yet unclear. The present study aimed at unraveling the molecular mechanisms underlying the pathogenesis of ROP. A comprehensive screening of candidate genes in preterms with ROP (*n* = 189) and no-ROP (*n* = 167) was undertaken to identify variants conferring disease susceptibility. Allele and genotype frequencies, linkage disequilibrium and haplotypes were analyzed to identify the ROP-associated variants. Variants in *CFH* (*p* = 2.94 × 10^−7^), *CFB* (*p* = 1.71 × 10^−5^), *FBLN5* (*p* = 9.2 × 10^−4^), *CETP* (*p* = 2.99 × 10^−5^), and *CXCR4* (*p* = 1.32 × 10^−8^) genes exhibited significant associations with ROP. Further, a quantitative assessment of 27 candidate proteins and cytokines in the vitreous and tear samples of babies with severe ROP (*n* = 30) and congenital cataract (*n* = 30) was undertaken by multiplex bead arrays and further validated by western blotting and zymography. Significant elevation and activation of MMP9 (*p* = 0.038), CFH (*p* = 2.24 × 10^−5^), C3 (*p* = 0.05), C4 (*p* = 0.001), IL-1ra (*p* = 0.0019), vascular endothelial growth factor (VEGF) (*p* = 0.0027), and G-CSF (*p* = 0.0099) proteins were observed in the vitreous of ROP babies suggesting an increased inflammation under hypoxic condition. Along with inflammatory markers, activated macrophage/microglia were also detected in the vitreous of ROP babies that secreted complement component C3, VEGF, IL-1ra, and MMP-9 under hypoxic stress in a cell culture model. Increased expression of the inflammatory markers like the IL-1ra (*p* = 0.014), MMP2 (*p* = 0.0085), and MMP-9 (*p* = 0.03) in the tears of babies at different stages of ROP further demonstrated their potential role in disease progression. Based on these findings, we conclude that increased complement activation in the retina/vitreous in turn activated microglia leading to increased inflammation. A quantitative assessment of inflammatory markers in tears could help in early prediction of ROP progression and facilitate effective management of the disease, thereby preventing visual impairment.

## Introduction

Retinopathy of prematurity (ROP) is a complex disease of the retina with a multi-factorial etiology and an early intervention has been observed to prevent irreversible vision loss in some of these prematurely born infants (1). Its incidence in developed countries with adequate neonatological facilities (like United States) is 19.88% (2) while it is slightly higher (~30%) for middle-income developing countries (3, 4). In India, approx. two million babies are at risk of developing ROP annually (4) with an overall incidence estimated to be around 45% (5, 6). Hence, ROP is one of the major causes of visual impairment in India. Lower gestational age (GA), lower birth weight (BW), and oxygen supplementation are the primary risk factors associated with ROP (7). It is a self-limiting disease with initial symptoms of avascular retina that progresses to abnormal growth of retinal vessels causing retinal detachment (8). Hypoxia in the avascular retina is considered to be the primary cause for neovascularization in ROP that further activates various cellular pathways such as HIF1α, eNOS/iNOS, and vascular endothelial growth factor (VEGF) signaling leading to abnormal neovascularization (9, 10). However, the detailed molecular mechanisms underlying neovascularization in ROP have not been elucidated yet. So far, few functionally relevant genes (*NDP, FZD4, LRP5, CFH, VEGF, ANGPT2, EPO, BDNF*, and *CETP*) have been associated with ROP in a small fraction of cases. But, many of these variants could not be replicated across different ethnicities (11–13). Further, their roles in risk predictions and disease management are yet to be determined.

The protein profiles in the vitreous have been utilized for studying the underlying pathology of the retina due to its proximity. This has largely been accomplished by analyzing the levels of erythropoietin, VEGF, and cytokines like interleukins (IL-6, IL-7, IL-10, and IL-15), Eotaxin, FGF basic, G-CSF, GM-CSF, IP-10, and RANTES in the vitreous to identify their potential as biomarkers for ROP progression (14–16). Interestingly, interleukin-7 (IL-7), monocyte chemotactic protein-1, and macrophage inflammatory protein 1 (MIP-1α and MIP-1β) levels were also found to be significantly elevated in the cord blood serum of ROP (17). Earlier, low serum levels of IGF-1 and VEGF were reported in preterm babies with severe ROP and low GA (18, 19). Thus, the studies on protein profiling and genetic associations of ROP could explain the susceptibility of some preterm babies progressing to severe ROP.

Our study is an attempt to comprehensively elucidate the genomic basis of ROP and identify the potential biomarkers for progression to severe stages. Since, no mutations were observed in the Norrin signaling genes in ROP in our earlier study (20), we explored genes involved in angiogenesis, growth, and development of the fetal retina, trans-endothelial migration, oxidative stress, inflammation, and neurodegenerative processes, in order to understand their role in ROP pathogenesis. We observed strong associations of ROP with the variants in *CFH, CFB, CXCR4, FBLN5*, and *CETP* genes along with increased levels of proteins in the extracellular matrix (ECM) and complement pathways in the vitreous of these babies. We observed the presence of the activated microglia/macrophages in the retina and vitreous. We further demonstrated the activated microglial cells under hypoxia expressed complement C3, VEGF, and IL-1β, thereby resulting in abnormal blood vessel proliferation in the ROP-affected eyes. We also evaluated the inflammatory proteins as potential biomarkers for ROP based on their expressions in the tear samples of the ROP patients.

## Materials and Methods

### Study Subjects

The study protocol adhered to the tenets of declaration of Helsinki and was approved by the Institutional Review Board (LEC02-14-029) of the L V Prasad Eye Institute (LVPEI). Preterm babies referred for further management from the neonatal intensive care units of different hospitals in Hyderabad to the LVPEI between January 2007 and December 2010 were enrolled. Overall, the study cohort comprised 372 preterm babies of GA ≤ 35 weeks and/or BW ≤ 1,700 g with ROP (*n* = 189) and no-ROP (*n* = 167). A detailed demographic and clinical history (Table S1 in Supplementary Material) of all the preterm babies enrolled were documented and a written informed consent was obtained from their parents. The diagnosis and categorization of ROP cases from mild to severe form was based on severity (stages 1–5), location (zones I, II, III), amount of disease (clock hours), and presence or absence of “plus” disease following ICROP guidelines (20, 21) (Figure S2 in Supplementary Material). Severe ROP includes progressive disease, which requires prompt treatment. It includes any stage (1–5) Zone I with plus and stages 2–3 Zone II with plus. Mild ROP cases include less severe disease, which does not require any treatment. Although until the regression of the disease completely, babies are under regular follow-up for ROP screening.

### Sample Collection

Venous blood (0.5–1 mL) was collected from the ROP and no-ROP preterm babies by venipuncture. DNA was extracted from the blood samples using an automated DNA extraction platform (MagNa Pure LC 20, Roche) following the manufacturers guidelines. Likewise, for proteomic studies, the vitreous humor samples (100–500 µL) were collected from preterm babies with stage IV and V ROP (*n* = 30) who had undergone vitrectomy as a part of their routine clinical management. The controls for the proteomic studies included babies with congenital cataract (<6 months of age) who underwent partial vitrectomy as part of the surgical management (*n* = 30). The vitreous samples were immediately centrifuged at relative centrifugal force (rcf) of 10,621 g and the supernatant was stored at −80°C deep freezer until further use.

Additionally, crude tears were collected before instilling any drops or drug for pupil dilation in the eyes of preterm babies with ROP (Stage II–V) (*n* = 27) and no-ROP (*n* = 13) using a capillary tube and without touching the conjunctiva. The tear samples for ROP subjects were collected during the active disease condition either before or after 3 months following medical intervention.

### Customized Genotyping of Candidate Variants

A customized panel containing 384 single-nucleotide polymorphisms (SNPs) from 26 chosen genes (Table S2 in Supplementary Material) involved in growth and development of the fetal retina, angiogenesis, inflammation, neurodegeneration, and oxidative stress processes were genotyped using a microarray platform (Illumina Inc., golden gate assay). Following hybridization, the fluorescent signals were scanned by a bead array reader and the raw signal intensities were imported to the Genome Studio software (version 1.9) for assessing quality scores. The assay and sample reliability were measured by means of the gen call score and the genotypes were called following clustering. The genotypes of a subset of samples for all the genes were validated by resequencing on an automated DNA sequencer (ABI 3130 XL) using the BigDye chemistry.

### Quantitative Assessment of Cytokines and Other Proteins in the Vitreous and Tear Samples

The concentrations of 27 cytokines (Bio-Plex Human cytokine 27-Plex, Bio-Rad, Hercules, CA, USA) and 28 different proteins (HMMP1-55K, HMMP2-55K, HNDG1-36K, HNDG2-36K, HTIMP2-54K, TGFB-64K-03, HYCYTOMAG-60K, Merck Millipore, Billerica, MA, USA) involved in ECM remodeling, angiogenesis and inflammatory pathways were screened by multiplex bead immunoassays using the Luminex xMAP technology in vitreous samples that were pre-diluted to concentration 1:3. Similar assay was used for estimating the concentrations of MMPs and cytokines in the tears samples and were quantitated by comparing them with their respective standard curve. All standards and some of the samples (due to less volume of samples) were measured in duplicates.

### Validation of the Differentially Regulated Proteins by Western Blotting and Zymography

A part of vitreous sample (50–100 µL) was lysed using a buffer containing 50 mM Tris-HCl (pH = 8), 120 mM NaCl, 0.5% NP40, protease inhibitor cocktail, and precipitated with acetone. The protein pellet was eluted in 50 µL of 1× phosphate buffered saline (PBS) containing the protease inhibitor cocktail and quantified by bicinchoninic acid assay. The normalized vitreous proteins (10 µg) were then subjected to western blotting. Western blotting was done using mouse anti-human C3 antibody (sc-28294, Santa Cruz) and mouse anti-human C5 (MAB2037, R&D Systems) followed by incubation with IRDye^®^ 680RD secondary antibody. The entire procedure was done according to the manufacturer’s recommended application protocol (https://www.licor.com).

MMP gelatinase activity was measured in the vitreous and tear of ROP babies and controls by zymography as described earlier (22). An equal volume of crude vitreous and tear samples were electrophoresed under non-reducing conditions in 10% SDS-PAGE gels polymerized with 1 mg/mL gelatin. The gel was washed with 2.5% Triton X-100 for 30 minutes at room temperature with gentle agitation, followed by rinsing with distilled water. The gel was then incubated for 30 min in developing buffer containing 50 mM Tris-HCl, pH 7.8, 5 mM CaCl_2_, 0.2 M NaCl, 0.02% Brij 35. The gel was incubated with fresh developing buffer at 37°C for 16 h and stained with Coomassie blue (Bio-Rad). This was followed by destaining with 10% v/v methanol, 5% v/v acetic acid in dH_2_O. Active MMP (MMP2/MMP9) band was detected in the zymogram in the discovery cohort. The observations of discovery cohort were further validated in tears in an extended cohort of patients at different stages of ROP: no-ROP (*n* = 9), mild ROP regressed (*n* = 6), mild ROP_progressed (*n* = 7), and severe ROP (*n* = 12) by zymography in order to confirm their role as biomarker in ROP pathogenesis. Zymogram band intensities were calculated with ImageJ software.

### Immunohistochemistry and Hematoxylin and Eosin (H&E) Staining for Macrophage/Microglia in the Vitreous

Vitreous were subjected to cytospin to separate the cells that were embedded in the parafilm block. Sections were cut, air dried, and stained with H&E (23) for understanding their morphology. Tissue sections were then deparaffinized using xylene and gradually rehydrated with ethanol. Antigen retrieval was done by microwaving the sections at full power for 4–5 min in Tris EDTA buffer (10 mM Tris, 1 mM EDTA, 0.5% tween 20, pH 9.0). Blocking was carried out with 2.5% (w/v) BSA in PBS (10 mmol/L sodium phosphate, pH 7.5, 120 mmol/L sodium chloride) for 30 min at room temperature. Thereafter, the slides were incubated for 60 min with the primary antibody (CD 68 for human 1:100) diluted in 1× PBS, followed by three washings with PBS. Further incubation was carried out with biotinylated anti-mouse immunoglobulin. Sections were then washed in PBS and incubated with avidin DH/biotinylated horseradish peroxidase reagent in PBS for 30 min before final washing. The antigen was localized using 1 mg/mL diaminobenzidene tetrahydrochloride (DAB; Sigma), 0.2% H_2_O_2_ in 50 mmol/L Tris–HCl, pH 7.6, which appeared as a brown end product. Sections were then counterstained with DAPI for nuclei staining.

### Response to Hypoxia by the Cultured Microglia Cells

The human microglial cell line (CHME3) was cultured in DMEM containing 10% FBS along with antibiotics penicillin and streptomycin. The confluent cells were trypsinized using 0.25% trypsin-EDTA. Hypoxic stress was introduced in the microglia cells by treating them with Cobalt chloride (CoCl_2_) at various concentrations from 100 to 250 µm. Briefly, around 15,000 cells were seeded on a six well plate and then serum deprived for 6 h, followed by treatment with CoCl_2_ for 24 h on attaining 70–80% confluency. The serum deprived cells in the same duration that were not treated for hypoxia were used as controls.

### Ca^2+^ Staining and Live Cell Imaging

The cells were washed in HBSS (Thermoscientific, Waltham, MA, USA) and then incubated with the calcium binding dye Flu-4 (diluted with HBSS 1:750) for 30 min. After washing the cells with HBSS three times, live cell imaging was performed for 10 min using an EVOS fluorescent microscope (Thermo Fischer Scientific, Waltham, MA, USA) under 20× magnification. The cytosolic calcium flux was measured using the change in Fluo-4 intensity over time for individual cells (Excitation: 494 nm Emission: 506 nm) (24).

### Semi-Quantitative PCR

The RNA from untreated and treated cells was extracted by Trizol method (25). The cDNA was prepared using iScript cDNA synthesis kit (Bio-Rad, CA, USA). Semi quantitative PCR was carried out using the specific primers (Table S3 in Supplementary Material) for *VEGF165, C3, HIF1*α, *BAX*, and *IL-1*β while β*-actin* was used as an endogenous control.

### Statistical and Bioinformatic Analysis

Allele frequencies of all the 384 variants were calculated by gene counting method along with odds ratio and 95% CI. A *p* value < 0.05 was considered to be significant. The associated allele and haplotype frequencies were further analyzed for statistical correction using Bonferroni and permutations tests (*n* = 10,000 permutations). Estimates of Hardy–Weinberg equilibrium (*p* > 0.001), linkage disequilibrium (LD), and haplotype frequencies were calculated using the Haploview software (version 4.2) (26).

Protein and cytokine levels in ROP and control samples were represented as bar plot (the mean ± SE) and box plot (median, interquartile range, and whiskers). Comparison of proteins and cytokines levels between ROP and controls vitreous/tears were calculated using the unpaired Student’s *t-*test. A *p*-value < 0.05 was considered to be statistically significant. Since the cytosolic calcium level does not follow a normal distribution, we performed the testing of equality in medians for control and hypoxic condition using Wilcoxon rank-sum test.

## Results

### Involvement of Genes in ROP

Of the 384 variants screened (Table S2 in Supplementary Material), 73 were removed from further analysis as they were either not in Hardy-Weinberg equilibrium in the controls (*n* = 16), were monomorphic (*n* = 44), or had a call rate < 97% (*n* = 13). Thus, 311 SNPs from 26 genes were finally analyzed for association with ROP. Among these 37 SNPs in 14 genes (*AGTR1, ANGPT2, C3, CFH, CFB, CXCR4, FBLN5, H2AFX, IHH, MMP2, TGF*β*1, CETP, VEGF*, and *TSPAN12*) exhibited significant association (*p* < 0.05) with ROP (Table 1). Additionally, 5/37 associated SNPs in *CFH, CFB, CXCR4, FBLN5*, and *CETP* genes withstood Bonferroni correction. Intriguingly, only the *CETP* variant (rs891141) conferred significant risk of ROP, while the variants across the other genes were protective (Table 1). Strong LD was observed across all the variants (except rs1831821) in *CFH* and rs891141 and rs289716 in *CETP* gene, while moderate LD was observed between rs891141 and rs289713 in *CETP* and rs2268002 and rs2284340 in *FBLN5* (Figure S1 in Supplementary Material).

**Table 1:** Association of gene variants with retinopathy of prematurity (ROP).

Genes screened	Single-nucleotide polymorphism (SNP) ID	Location	Nucleotide change	Amino acid change	RegulomeDB (binding score) (27)	Minor allele	Minor allele frequencies		*p-*Value	Odds ratio [95% CI]
							ROP	Controls		
*CFH*	**rs374896**	**Intron**	g.71371T>C	–	Minimum binding evidence (6)	**T**	**0.0426**	**0.159**	**2.94 × 10^−7^**	**0.241 [0.135–0.431]**
*CFB*	**rs1048709**	**Exon**	g.19461A>G	p.R150R	Likely to affect binding of POLR2A and linked expression of the HLAC, HLA-DQA1, HLADQB1, HLADRB1, HLADRB5	**G**	**0.15**	**0.269**	**1.71 × 10^−5^**	**0.484 [0.035–0.676]**
*C3*	rs344550	Intron	g.37710G>C	–	Likely to affect binding of GATA2, MYC, NR2F2, STAT5A, SPI1CCNT2 (1F)	C	0.223	0.29	0.0409	0.703 [0.501–0.986]
	rs2287846	Intron	g.24106G>C	–	Minimal binding evidence (5)	G	0.298	0.237	0.0658	1.370 [0.979–1.915]
*CXCR4*	**rs2228014**	**Exon**	g.2652C>T	p.I142I	Minimum binding evidence (4)	**G**	**0.422**	**0.637**	**1.32 × 10^−8^**	**0.416 [0.307–0.565]**
*ANGPT2*	rs2922889	Intron	g.119194A>T	–	Minimum binding evidence (6)	T	0.497	0.431	0.0776	1.305 [0.971–1.756]
	rs2515464	Intron	g.35092A>C	–	Minimum binding evidence (5)	T	0.173	0.251	0.0102	0.622 [0.432–0.895]
	rs734701	Intron	g.32684C>T	–	Minimum binding evidence (6)	A	0.452	0.527	0.0465	0.741 [0.551–0.996]
	rs2959812	Intron	g.29629T>C	–	Minimum binding evidence (5)	C	0.471	0.548	0.0391	0.732 [0.544–0.985]
*VEGF*	rs2010963	5′-UTR	–	–	Minimum binding evidence (4)	C	0.302	0.243	0.0084	1.352 [0.969–1.888]
	rs1413711	Intron	g.2758T>C	–	Minimum binding evidence (4)	T	0.39	0.482	0.014	0.688 [0.51–0.928]
	rs1005230	Intergenic	–	–	Minimum binding evidence (5)	A	0.388	0.482	0.011	0.680 [0.505–0.917]
*FBLN5*	**rs2268002**	**Intron**	g.17582G>C	–	Minimum binding evidence (5)	**G**	**0.32**	**0.423**	**9.2 × 10^−4^**	**0.641 [0.492–0.835]**
*MMP2*	rs2285052	Intron	g.88546A>C	–	Minimum binding evidence (5)	G	0.092	0.036	0.0025	2.732 [1.39–5.368]
*TGFb1*	rs11466359	Intron	g.22217C>T	–	Minimum binding evidence (5)	T	0.093	0.0482	0.021	2.027 [1.1–3.734]
	rs4803457	Upstream	g.4544T>C	–	Minimum binding evidence (4)	A	0.465	0.536	0.061	0.754 [0.561–1.013]
*CETP*	**rs891141**	**Intron**	g.7962G>T	–	Minimum binding evidence (5)	**C**	**0.234**	**0.114**	**2.99 × 10^−5^**	**2.378 [1.57–3.598]**
*H2AFX*	rs640603	Intergenic 3′ of a gene	–	–	Likely to affect binding of PLR2A, CHD1, E2F6, MXI1, E2F4, E2F6, MYC (2b)	T	0.161	0.09	0.0049	1.940 [1.215–3.085]
*TSPAN12*	rs41624	Intron	g.62819A>G	–	Minimum binding evidence (6)	T	0.176	0.243	0.0279	0.665 [0.462–0.958]
	rs41629	Intron	g.59568T>G	–	Minimum binding evidence (6)	A	0.176	0.243	0.0279	0.665 [0.462–0.958]
	rs3735467	Intron	g.47721G>T	–	Minimum binding evidence (5)	C	0.177	0.246	0.031	0.669 [0.465–0.964]
	rs12669167	Intron	g.38926T>G	–	NA (7)	C	0.173	0.24	0.0279	0.664 [0.46–0.958]
	rs10225453	Intron	g.36511C>A	–	Minimum binding evidence (5)	T	0.17	0.236	0.0287	0.663 [0.458–0.959]
	rs6953454	Intron	g.33908G>A	–	NA (7)	T	0.172	0.237	0.03	0.666 [0.461–0.964]
	rs996903	Intron	g.32898A>G	–	Likely to affect binding and linked expression of the FLJ21986 (1F)	C	0.17	0.237	0.0279	0.662 [0.458–1.044]
	rs6959328	Intron	g.32490T>A	–	NA (7)	A	0.17	0.237	0.0279	0.662 [0.458–1.044]
	rs6466759	Intron	g.28767A>T	–	Minimum binding evidence (5)	T	0.17	0.238	0.0251	0.657 [0.454–0.95]
	rs7805211	Intron	g.25107G>A	–	Minimum binding evidence (6)	A	0.168	0.237	0.0218	0.65 [0.449–0.941]
	rs6466760	Intron	g.24403C>G	–	Minimum binding evidence (6)	C	0.168	0.237	0.0218	0.65 [0.449–0.941]
	rs6466762	Intron	g.16639G>A	–	Minimum binding evidence (5)	A	0.169	0.241	0.0167	0.638 [0.441–0.923]
	rs3823859	Intron	–	–	Minimum binding evidence (5)	G	0.17	0.24	0.0219	0.651 [0.451–0.941]
	rs17142995	Intron	g.11660A>G	–	Minimum binding evidence (6)	C	0.173	0.24	0.0279	0.664 [0.46–0.958]
	rs7781985	Intron	g.6475A>C	–	Likely to affect binding and linked expression of the FLJ21986/monocytes (1F)	C	0.17	0.24	0.0219	0.651 [0.451–0.941]
	rs3757557	5′UTR	g.91G>A	–	Minimum binding evidence (4)	A	0.117	0.171	0.0411	0.644 [0.421–0.985]
	rs4141309	Intergenic, upstream 5′ of gene	–	–	Minimum binding evidence (4)	A	0.112	0.171	0.0236	0.611 [0.398–0.939]
*AGTR1*	rs2739504	Intron	g.13100A>G	–	Minimum binding evidence (4)	C	0.446	0.371	0.041	1.362 [1.012–1.833]
*IHH*	rs394452	Exon	g.5153T>C	p.T376T	Minimum binding evidence (5)	T	0.261	0.168	0.0027	1.75 [1.211–2.528]

*SNPs in bold withstood Bonferroni correction*.

Likewise, haplotypes generated with the associated and flanking variants of these five genes revealed that only the haplotype C-A-T in *CETP* conferred significant risk of ROP, while those with *CFH* and *FBLN5* were protective. Haplotypes with the *CXCR4* and *CFB* were not informative (Table 2). Thus, the present study highlights the potential involvement of novel genes (*CFH, CFB, CETP, FBLN5*, and *CXCR4*) in ROP based on their allelic and haplotype associations.

**Table 2:** Estimated haplotype frequencies of the significantly associated variants in *CETP, CFH*, and *FBLN5* genes in retinopathy of prematurity (ROP) and premature controls.

Genes (single-nucleotide polymorphisms)	Haplotypes	Overall frequencies	ROP frequencies	Controls frequencies	Chi square	*p-*Value	Odds ratios (95% CI)
*CETP* (rs891141, rs289713, rs289716)	A-A-T	0.327	0.313	0.343	0.734	0.3916	0.871 (0.637–1.193)
	A-A-A	0.3	0.294	0.308	0.156	0.6931	0.937 (0.68–1.292)
	C-A-T	0.149	0.191	0.101	11.358	0.0008	2.1 (1.354–3.256)
	A-T-A	0.143	0.121	0.169	3.357	0.0669	0.674 (0.442–1.029)
	A-T-T	0.053	0.04	0.067	2.488	0.1147	0.586 (0.3–1.144)

*CFH* (rs3753395, rs374896, rs393955*)*	T-C-T	0.563	0.578	0.547	0.693	0.4052	1.134 (0.843–1.526)
	A-C-G	0.188	0.233	0.139	10.066	0.0015	1.874 (1.268–2.769)
	A-C-T	0.151	0.149	0.154	0.032	0.8586	0.962 (0.638–1.451)
	A-T-G	0.096	0.04	0.156	27.61	1.48 × 10^−7^	0.226 (0.125–0.41)

*FBLN5* (rs2268002, rs2284340)	G-C	0.38	0.413	0.343	3.621	0.0571	1.346 (0.992–1.826)
	G-G	0.37	0.311	0.437	12.093	5 × 10^−4^	0.58 (0.427–0.789)
	A-C	0.242	0.271	0.21	3.625	0.0569	1.4 (0.989–1.983)

### Quantitative Assessment of Proteins Involved in Complement Cascade and Neurodegeneration in the Vitreous Samples of ROP Subjects

**Figure 1:**
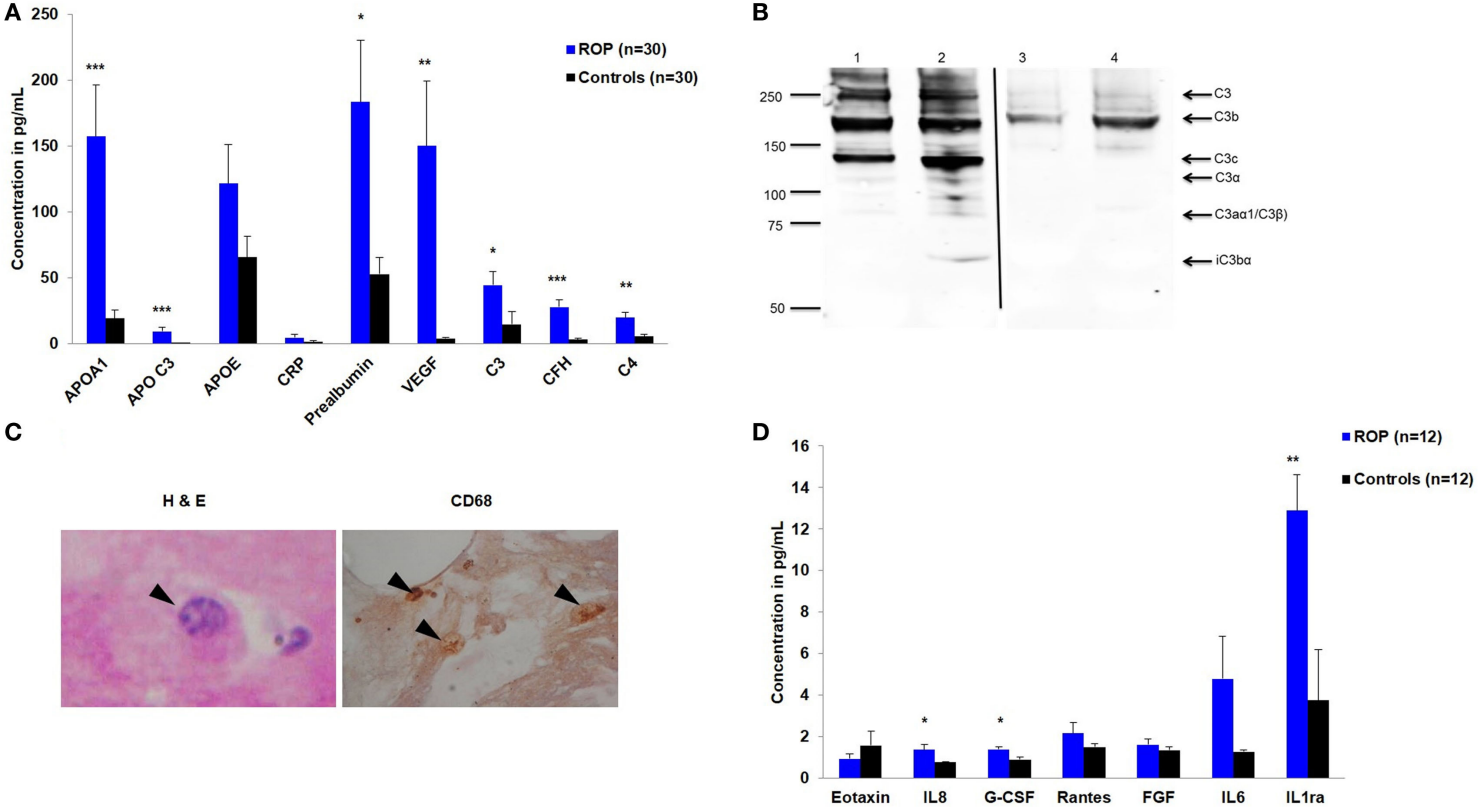
Expression of inflammatory molecules in proliferative retinopathy of prematurity (ROP). **(A)** Differential levels of complement components and apolipoproteins in the vitreous humor of ROP and controls **(B)** Western blotting of C3 done under non-reducing condition, showing its expression in patients (lane 1 and 2) and controls (lane 3 and 4). Arrows indicate the C3 fragments. Thin black line inserted within gel showing that all the lanes were run on the same gel but were not continuous **(C)** Hematoxylin and eosin staining of ROP vitreous. Arrowheads indicate degenerated morphology of macrophages at 40×-magnification (irregular shaped with large vacuole, nucleus with a very prominent nucleolus); Staining of ROP vitreous with CD68 marker. Arrowheads indicate the presence of activated macrophages at 10× magnification. **(D)** Differential levels of inflammatory cytokines in the vitreous humor of ROP and controls. Error bars in **(A)** and **(D)** show SEM, *p < 0.05, **p < 0.005, ***p < 0.0005 (ROP vs. controls).

Based on strong associations in the *CFH* and *CFB* genes, a quantitative assessment of a neurodegenerative panel containing CRP, SAP, MIP-4, Complement C4, apolipoprotein AI, apolipoprotein CIII, apolipoprotein E, Complement Factor H, and Complement C3 proteins was carried out by multiplex immuno-bead assay in the vitreous samples of ROP patients (*n* = 30) and controls (*n* = 30). All the complement components and apolipoproteins were detectable in the vitreous samples. Overall, we observed significantly elevated levels of C3 (*p* = 0.05), C4 (*p* = 0.001), CFH (*p* = 2.24 × 10^−5^), VEGF (*p* = 0.0027), apolipoprotein AI (*p* = 0.0007), and apolipoprotein CIII (*p* = 0.004) in the vitreous of ROP compared to the control subjects indicating their possible involvement in the disease pathogenesis (Figure 1A).

### Activation of Complement Pathway in Vitreous Humor of Proliferative ROP

We validated the differential expression of complement component C3 by western blotting. An intense band of 192 kDa corresponding to C3 molecule was observed in ROP cases compared to controls (Figure 1B). Additionally, we observed the activated C3 fragments; C3b (182 kDa), C3c (145 kDa), and iC3bα (63 kDa) in the ROP vitreous under non-reducing conditions as confirmed by mass spectrometry (data not shown here), but not in the controls (Figure 1B), suggesting a higher activation of C3 in ROP. Likewise, a higher expression of complement component C5 was observed in ROP vitreous compared to the controls (data not shown) suggesting a further activation of the complement pathway.

### Hypoxia-Induced Activated Macrophage Secretes Angiogenic Molecules

We further demonstrated that along with increased expression of angiogenic molecules in the vitreous samples of patients, activated macrophages/microglia in turn would also be secreting proinflammatory cytokines that might exacerbate the inflammation, further playing a role in the ROP pathogenesis. We detected activated macrophages/microglia on H&E, further confirmed by immunostaining with CD68 in ROP vitreous but not in the controls (Figure 1C). The results of this experiment supported for shift in the proangiogenic state as demonstrated by a significant increase in the levels of cytokines IL8 (*p* = 0.0149*)*, G-CSF (*p* = 0.0099*)*, IL1ra (*p* = 0.0019), and VEGF (*p* = 0.0027*)* (Figures 1A,D) along with marginal increase of IL6, IL12, IL7, RANTES, and MCP1 in the ROP vitreous (data not shown).

**Figure 2:**
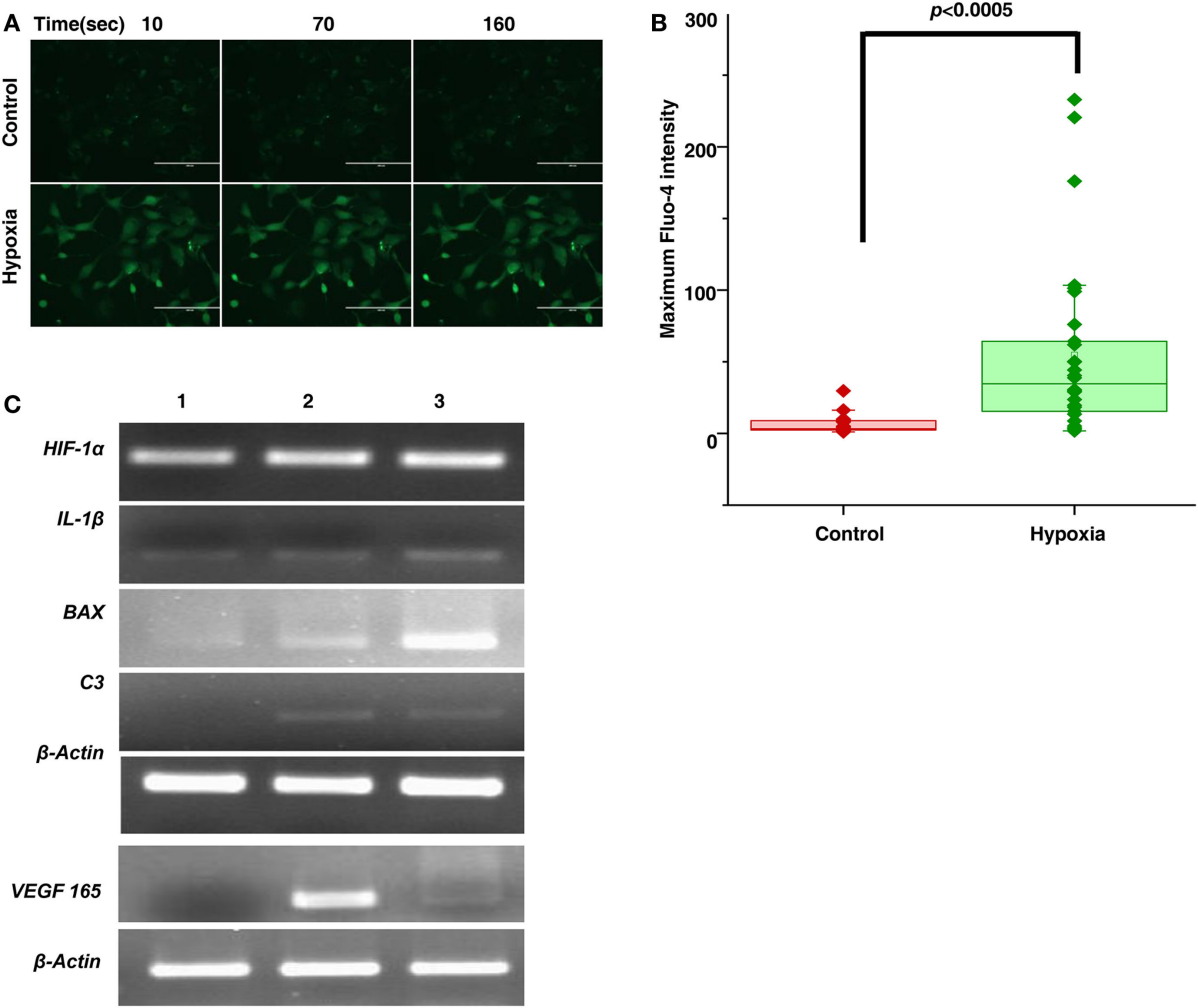
Hypoxia induced microglia secretes inflammatory and angiogenic molecules. **(A)** Time lapse imaging of cytosolic calcium in microglial cells in normal and hypoxic conditions. Scale bar: 200 µm **(B)** Comparison of Ca_max_ (Maximum Fluo-4 intensity) in microglial cells for control and hypoxic condition [Ca^2+^transients were measured for 50 cells (*n* = 50) for each cases]. Data are presented as box plot and were analyzed using Wilcoxon rank-sum test. **(C)** Semi-quantitative PCR was used to evaluate the expression of secreted product (*HIF-1*α, *IL-1*β, *BAX, C3, VEGF*) of hypoxia-induced microglial cells treated with no CoCl_2_ (lane 1), 100 µM CoCl_2_ (lane 2), and 150 µM CoCl_2_ (lane 3) and β-actin as control.

To further confirm these results, we subjected the cultured microglial cells to hypoxic condition and checked for the expression of proinflammatory markers. The effect of hypoxia on the activation of macrophages/microglia was observed with an intense calcium staining in cells exposed to hypoxia compared to the unexposed ones. The result shows that there is increase in cytosolic calcium levels in case of hyperactivated cells subjected to 24 h of hypoxic stress (Figure 2A, *n* = 50). Specifically, there is a significant increase (*p* < 0.0005) in cytosolic calcium (Ca_max_ = the maximum Fluo-4 intensity) in microglial cells followed by hypoxia exposure (Figure 2B). Likewise, a higher expression of complement *C3, VEGF165*, and hypoxia inducing factor-1α (*HIF-1*α) was also observed in exposed cells (Figure 2C).

### Involvement of Extra-Matrix Metalloproteinases in Pathogenesis of ROP

**Figure 3:**
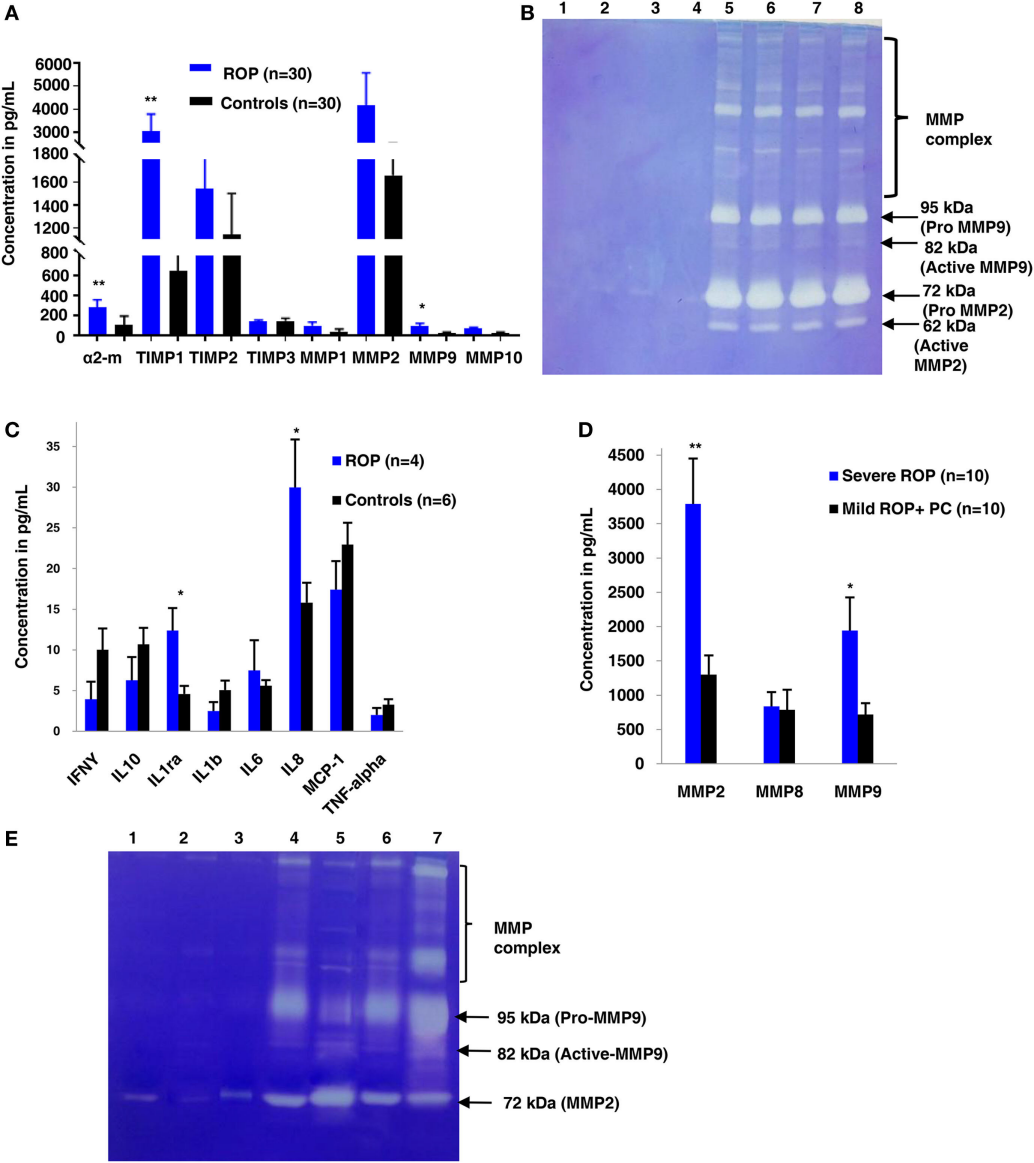
Extracellular matrix (ECM) metalloproteinases and cytokines increases in proliferative retinopathy of prematurity (ROP) in both vitreous and tears samples. **(A)** Differential levels of ECM proteins and its inhibitors in ROP and control vitreous [*p < 0.05, **p < 0.005, *** *p* < 0.0005 (ROP vs. controls)]. **(B)** Zymogram shows activation of MMPs in ROP vitreous (lanes 5, 6, 7, 8) as compared to controls (1, 2, 3, 4). **(C)** Differential levels of cytokines in ROP (5 µL) and control tears (5 µL). **(D)** Differential levels of MMPs in ROP (5 µL) and control tear (5 µL) [*p < 0.05, **p < 0.005, ***p < 0.0005 (severe ROP vs. mild ROP + premature controls)]. **(E)** Zymography showing more activation of MMPs in severe ROP (lanes 6, 7) as compared to mild ROP (lanes 4,5) and controls (5 µL tears; lanes 1, 2, 3). Error bars show SEM.

A strong association of SNPs in *FBLN5* and moderate association of *MMP2, TGF*β gene (Table 1) with ROP suggested the role of ECM proteins in ROP pathogenesis. Further, a quantitative assessment of the ECM proteins indicated a significant increase in MMP9 (*p* = 0.038), TIMP1 (*p* = 0.004), and α2 macroglobulin (*p* = 0.0018) in the ROP vitreous (Figure 3A). We also assessed the MMP activation in ROP by gelatine zymography. Our results showed higher levels of both pro and activated MMPs (MMP9 and/or MMP2) in the vitreous of patients suggesting its potential role in disease pathogenesis (Figure 3B).

### Exploring the Potential of Inflammatory Markers in Tear Samples for the Progression of ROP

**Figure 4:**
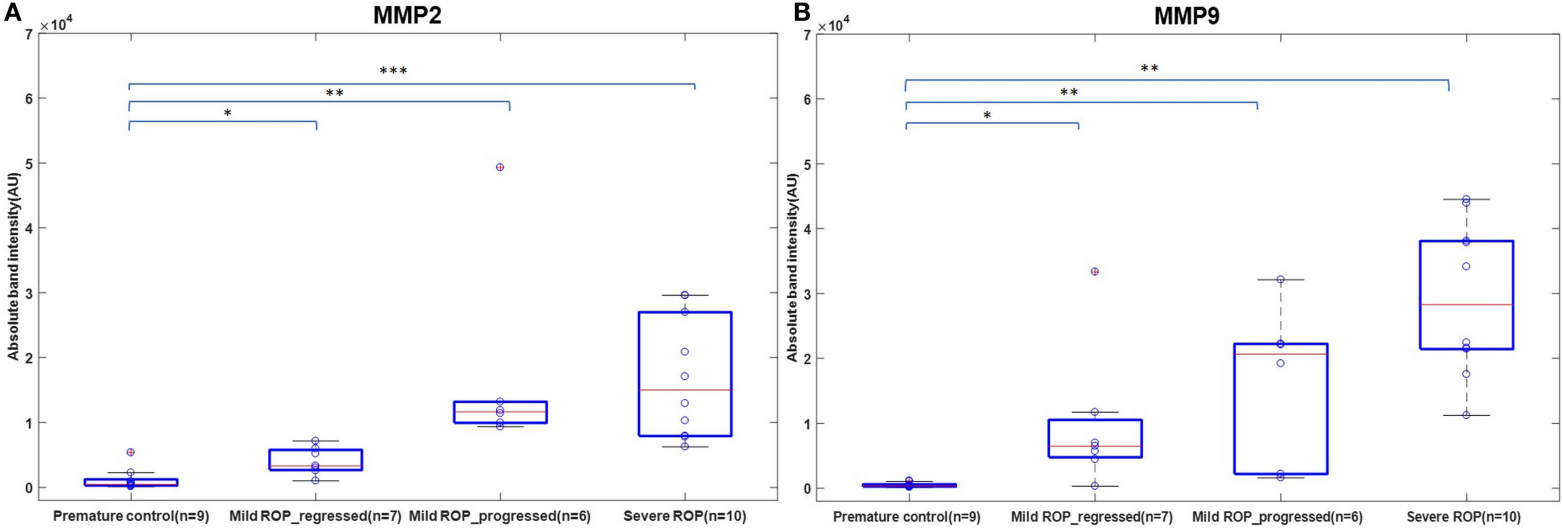
A role of MMPs in tears as biomarker for retinopathy of prematurity (ROP) progression: differential expression of Zymogram band intensities of **(A)** MMP9 and **(B)** MMP2 in extended cohort including severe ROP (*n* = 10), mild ROP_progressed (*n* = 6), mild ROP_regressed (*n* = 7), and no ROP premature controls (*n* = 9) [*p < 0.05, **p < 0.005, ***p < 0.0005 (ROP vs. controls)], AU, arbitrary unit.

We explored if increased expression of inflammatory markers (as seen in the vitreous samples of ROP patients) could also be reproducibly detected in tears and further be established as the biomarker for disease progression. A quick multiplex ELISA of tear samples collected from the ROP babies at different stages and no-ROP preterms was performed for some inflammatory markers (interleukins, TNFα, IFNγ, and MMPs). Significantly higher expressions of IL-1ra (*p* = 0.014), MMP2 (*p* = 0.0085), and MMP-9 (*p* = 0.03) were detected in severe ROP cases compared to mild ROP and no-ROP tear samples that was further confirmed by zymography (Figures 3C–E). On the zymogram, the tear samples from no-ROP showed very low expression of MMPs as compared to severe ROP (Figure 3E). These results were confirmed to be reproducible in the extended cohort of ROP with a significant increased expression of activated MMP2 in severe ROP (*p* = 0.0023) and progressive ROP (*p* = 0.007) as compared to mild ROP (*p* = 0.01) and premature controls. Similar pattern of gradual increase in MMP9 expression was also observed in mild ROP (*p* = 0.02) to progressive (*p* = 0.001) and severe ROP cases (*p* = 1.2 × 10^−6^) with respect to premature controls (Figure 4).

## Discussion

Retinopathy of prematurity is a biphasic disease that includes an initial phase of hyperoxia leading to blood vessel obliteration followed by hypoxia causing vessel proliferation eventually leading to neovascularization and neurodegeneration. It is a complex disease with multifactorial etiologies. An earlier study on monozygotic and dizygotic twin pairs had also noted the genetic involvement in the development of ROP, in absence of other environmental factors (28). While supplemental oxygen is considered as a major risk factor along with lower GA and BW, studies from India and other Asian countries have reported ROP babies with higher GA and BW (29) and oxygen supplementation does not always predict the risk of ROP (6). Therefore, we hypothesized that genetic predisposition along with environmental/maternal or other risk factors may lead to the development of ROP.

A strong association of gene variants involved in the complement pathway (*CFH, CFB, C3*), ECM remodeling (*FBLN5, MMP9*), leukocyte transendothelial migration and activation (*CXCR4*), HIF1A signaling and angiogenesis (*ANGPT2, H2AFX*, and *VEGF*), and developmental processes (*TGFb1, IHH*) observed in the present study (Table 1), confirms the involvement of genes in ROP pathogenesis. A previous study reported the association of polymorphisms in *IHH, AGTR1, TBX5, CETP, GP1BA, EPAS1, BDNF*, and *CFH* with ROP (11, 13). However, only a few of these associated variants could be replicated in the present cohort, indicating allelic heterogeneity (Table 3). Thus, the novel and associated variants identified in the present study (Tables 1 and 3) and elsewhere should be screened across multiple populations to understand their implications in ROP.

**Table 3:** Comparison of commonly associated gene variants in retinopathy of prematurity worldwide.

Associated genes	Associated single-nucleotide polymorphisms (associated allele)	Present study (India), ^a^189/167	Study by Mohamed et al. (11) (U.K.), ^a^102/228	Study by Hartnett et al. (13) (USA), ^a^593/364
*CFH*	rs529825 (A)	*p* = 0.7521	*p* = 0.01	–
	rs800292 (A)	*p* = 0.3186	*p* = 0.01	–
	rs379489 (A)	*p* = 0.4343	–	*p* = 0.3926
	rs395544 (A)	*p* = 0.5525	–	*p* = 0.403
*CETP*	rs289747 (T)	*p* = 0.5688	*p* = 0.004	–
*GP1BA*	rs2243093 (C)	*p* = 0.2991	*p* = 0.005	–
*TBX5*	rs1895602 (T)	*p* = 0.352	*p* = 0.003	–
*AGTR1*	rs33978228 (G)	*p* = 0.0196	–	–
	rs427832 (G)	*p* = 0.2177	*p* = 0.005	–
*IHH*	rs3099 (C)	*p* = 0.1565	*p* = 0.003	–
*EPAS1*	rs1867785 (G)	*p* = 0.958	*p* = 0.001	–

*^a^Cases/controls*.

**Figure 5:**
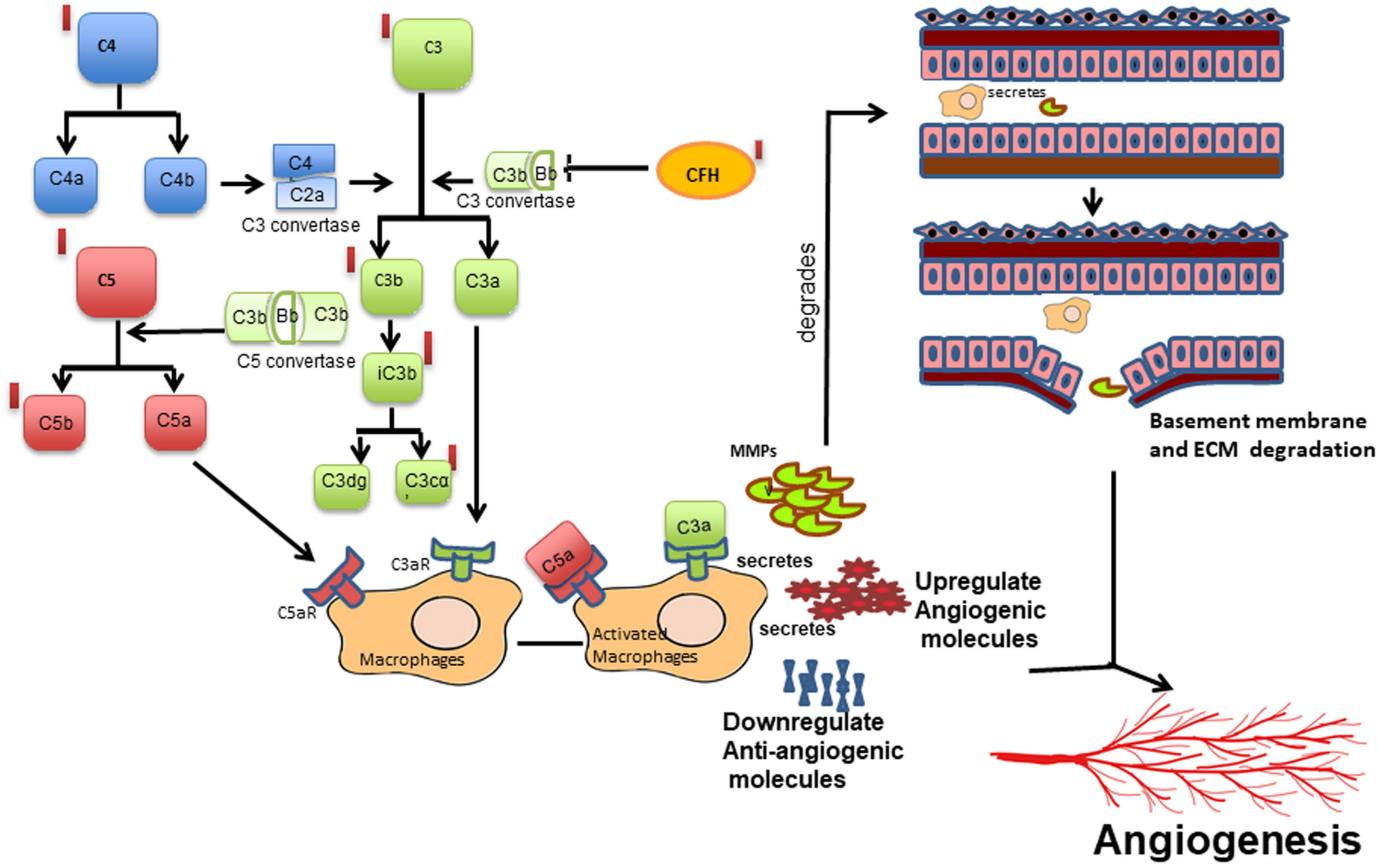
Schematic diagram of the proposed mechanism of complement activation and its potential effect on macrophage-mediated angiogenesis in retinopathy of prematurity. Cleavage of complement components C3 into C3a, C3b and further into iC3b, C3dg and C3cα; C4 into C4a and C4b and C5 into C5a and C5b leading to the activation of complement cascade, which in turn activates macrophage/microglia or *vice versa*. Activated macrophage/microglia secretes MMPs, cytokines, proangiogenic proteins, and reduced angiogenesis inhibitors that may lead to increased vessel proliferation and extracellular matrix (ECM) degradation in turn promoting angiogenesis. Red bars represent upregulation of the complement components observed in the present study.

The strong associations of *CFH, CFB*, and *C3* variants in our ROP patients along with elevated levels of C3 and CFH proteins in their vitreous (Table 1 and Figure 1A) indicated a possible involvement of the alternative complement pathway in ROP. CFH and CFB are the regulators of the alternative complement immune pathway (30). Upon activation, CFB is cleaved by complement factor D yielding two subunits, Ba and Bb. The active subunit Bb associates with C3b to form C3 convertase of alternative pathway while CFH regulates the alternative pathway activation by accelerating the decay of C3 convertase (30). It was also noted that there was an increase in the formation of CFB in oxygen induced retinopathy (OIR) mice model (31). Thus, the observed genetic associations of *CFH* and *CFB* complemented with their increased expression of cleaved C3 protein fragments in the vitreous of ROP-affected eyes in our study confirmed their possible involvement in disease pathogenesis (Figure 5). Generally, complement factors are known to be downregulated in the normal preterm neonates because of immature development of the immune system (32, 33). On the contrary, we observed an elevation and activation of the complement components and complement factors in the vitreous of ROP patients at infancy (Figure 1), suggesting an important role of the complement pathway in ROP pathogenesis.

Interestingly, the genetic variants in *CFH, C3*, and *CFB* genes have also been associated with AMD susceptibility (34, 35). A Y402H variant in the *CFH* gene was found to be most strongly associated with AMD patients worldwide. However, the ROP-associated *CFH* variant (rs374896) identified in the present study is located in the intron of the gene (Table 1). Further, functional studies on CFH in AMD eyes have shown that chronic low grade intraocular complement activation in patients carrying the risk variants in *CFH* along with exposure to environmental triggers (smoking, oxidative stress, etc.) causes the retinal pigment epithelial damage leading to neurodegeneration and neovascularization and eventually visual loss (36).

Complement components do not mediate neovascularization by itself but *via* the inflammatory cells (37). As was demonstrated in an OIR mouse model, complement factors C3a and C5a activate macrophages by binding to C3aR and C5aR, thereby regulating angiogenesis (38). In the present study, a strong association (*p* = 1.32 × 10^−8^) of rs2228014 in *CXCR4* (Table 1) along with the presence of activated microglia/macrophages in the vitreous (Figure 1C), implicate their role in ROP angiogenesis via the leukocyte transendothelial migration. CXCR4 is a chemokine receptor for stromal derived factor 1 (CXCL12/SDF-1), which is mainly involved in the extravasation and migration of lymphocytes and monocytes (39). Inhibition of CXCR4 has been shown to result in reduced vascular sprouting following VEGF treatment in retinal explants (40). Our observation of the association of variants in *VEGF, ANGPT2*, and *H2AFX* (Table 1) indicate the involvement of HIF1α signaling pathway (hypoxia) in ROP pathogenesis. Furthermore, the secretion of C3 and VEGF by microglial cells under hypoxia (Figure 2) validated that hypoxia induced microglia/macrophage along with the complement component, could be contributing to the neovascularization in ROP eyes. The high level of α2-macroglobulin in the ROP vitreous (Figure 3A) also indicated the presence of activated macrophages/microglia that further interacts with low-density lipoprotein receptor-related protein 1 (LRP1) which in turn induces MMP9 expression (41, 42).

Based on published studies on macrophages/microglia activation leading to inflammation (37), we proposed that increased expression of the complement components, VEGF, other cytokines, and ECM components (MMPs) in the vitreous of ROP patients are mediated by macrophage/microglia activation by creating an imbalance of angiogenic and anti-angiogenic molecules (Figure 5). The proteolytic degradation of ECM is a critical step for the invasion of blood vessels during neovascularization. MMPs are endoproteases that cleaves the protein components of the ECM while TIMPs, α2 macroglobulins, and α1 antitrypsin are the proteinase inhibitors (43). In proliferative diabetic retinopathy, the elevated levels of MMP-2 and MMP-9 were shown to cause ECM remodeling (44, 45) further leading to net collagen IV degradation and vitreous liquefaction (46). The presence of activated MMP-9 in the vitreous along with elevated levels of TIMP1, TIMP2, and α2 macroglobulin in our ROP patients and presence of blood component proteins like apolipoproteins (Figures 1A and 3A) explained its role in the degradation of the basement membrane of blood vessels, seeping out into the vitreous along with the other blood components, thereby causing vitreous hemorrhage and vitreous liquefaction.

Presence of inflammatory markers in the vitreous or other body fluid in young preterm babies might suggest an infectious etiology and inflammatory stimuli contributing to ROP (47). Fetal inflammatory response syndrome (including sepsis, periventricular leukomalacia, intraventricular hemorrhage, necrotizing enterocolitis, and bronchopulmonary dysplasia), chorioamnionitis, and microbial infections are some of the predisposing factors for inflammation observed in some studies (48). There was no evidence of exposure to infection in our cohort since babies with any microbial infections were excluded. Additionally, we did not find any difference in the complement levels or activation patterns in the serum of these patients and controls unlike in the vitreous samples, further ruling out any systemic infection (data not shown). Based on these evidences, neonatal non-infectious inflammation might be playing a major role in the pathogenesis of ROP.

Based on these findings supplemented with increasing evidences on the role of inflammation in causing neovascularization, we speculated if MMPs could be detected in the tear samples of ROP babies so that it could be used as markers for ROP progression. The tear samples were an obvious choice for this study as it is fairly non-invasive, safe, and convenient, although there were some restrictions of tear volume and sampling in the ROP babies. It was interesting to note that the levels of MMPs in tears were significantly higher in severe ROP compared to no-ROP and mild ROP eyes that underscored its potential use as a biomarker for an early prediction of this condition (Figures 3E and 4). This was further confirmed by zymography, with an increasing trend of the activated MMPs (both 2 and 9) in all the samples of severe stages of ROP along with a case of mild ROP. This mild ROP baby eventually progressed very quickly to a severe stage (plus ROP) in 2 weeks and did not respond to laser therapy (Figure 3E). The subsequent validation of these initial findings was done in an extended cohort and the increased levels of MMPs with the increase in severity of disease further established the usefulness of MMPs in tears as potential biomarkers. While our data proved that the levels of MMPs could reliably predict the progression of ROP (Figure 4), we could not perform any longitudinal analysis of MMPs levels in the tears due to the difficulties in obtaining samples from preterm babies at regular intervals. Likewise, a direct correlation of genotypes with protein levels or their activities in the corresponding biological material (vitreous/aqueous/tear) and clinical phenotype could not be attempted. Nevertheless, our study provided a proof of concept that tear MMPs levels could be a potential predictor for ROP progression in preterm babies.

In conclusion, the assessment of the activation of alternate complement pathway in ROP based on the novel genetic associations indicated the possible mechanisms of immune activation that could lead to aberrant neovascularization in the retina. However, the detailed underlying mechanisms of immune activation in abnormal blood vessel proliferation and neurodegeneration in the early stages of ROP are yet to be understood. Additionally, our results emphasized the primary role of complement component C3 in abnormal angiogenesis as seen in proliferative ROP. The proteins involved in the alternative complement pathway could be targeted selectively to prevent neovascularization, which might be helpful in preventing vision loss due the progression of ROP. The association of ECM-related genes with ROP along with elevated levels of the corresponding ECM proteins and its activation in the vitreous of ROP patients suggested its possible role in blood-retinal barrier degradation, which could promote neovascularization. Finally, the elevated levels of MMPs in tears of ROP patients established its role as a potential biomarker for the prediction of progression to proliferative stages. However, this needs to be replicated in other extended cohorts worldwide using a longitudinal study design. The present treatment strategies for managing severe ROP are inefficient as they target only the later vasoproliferative phase of ROP. Diagnosing and treating the disease at an earlier stage would definitely help in the timely and efficient management of this disease. The results of this study would aid in finding biomarkers for predictive testing as well as identifying newer drug targets for an efficient management of ROP.

## Ethics Statement

The study protocol adhered to the tenets of declaration of Helsinki and written informed consent was obtained from the parents of all the minor subjects and was approved by the Institutional Review Board (LEC02-14-029) of the L V Prasad Eye Institute (LVPEI).

## Author Contributions

IK and SJ conceived the idea; IK, SJ, and SC wrote the protocol; IK served as principal investigator; SC, SJ, DB, RK, LG, PR, and PC were co-investigators; SJ, DB, RK, PR, and PC performed clinical examinations, graded the fundus images and did surgeries for the preterm and full term babies; SR, SP, and GM collected blood, vitreous and documented family history in the predesigned questionnaires; SR performed most of the molecular biology based analysis of blood and vitreous; SP performed the tear analysis; SSh performed cell biology work; LG and SS performed analysis for the Ca^2+^ imaging data; SR, IK, and SC analyzed the data and wrote the manuscript; and all authors revised the paper and approved the submitted version.

## Conflict of Interest Statement

The authors declare that the research was conducted in the absence of any commercial or financial relationships that could be construed as a potential conflict of interest.
