# Supplementary material for: Abnormal Complement Activation and Inflammation in the Pathogenesis of Retinopathy of Prematurity
Source: Front Immunol. 2017 Dec 22;8:1868. doi: 10.3389/fimmu.2017.01868 (PMC5743907; doi:10.3389/fimmu.2017.01868)
Supplement: Supplementary file 1 [file Image_1.PDF]

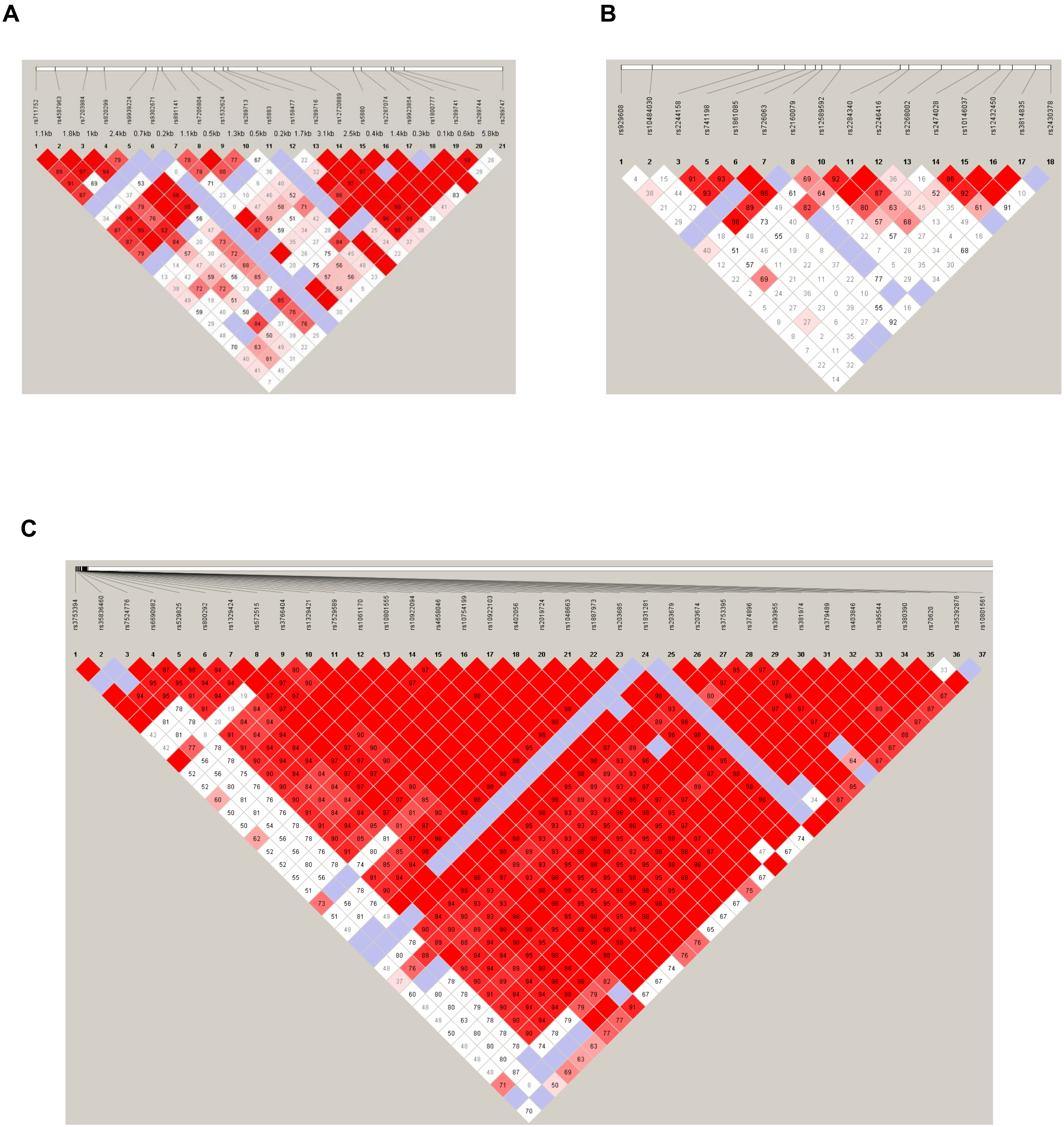

Supplementary figure 1: LD plot for A) *CETP* B) *FBLN5* C) *CFH* SNPs: Each square in a triangle plots the level of Linkage disequilibrium (LD) between a pair of sites in a region. Red coloring indicates strong LD, white coloring indicates weak LD. The long line indicates physical length of the region and short black line plot the position of each marker.
