# Supplementary material for: Abnormal Complement Activation and Inflammation in the Pathogenesis of Retinopathy of Prematurity
Source: Front Immunol. 2017 Dec 22;8:1868. doi: 10.3389/fimmu.2017.01868 (PMC5743907; doi:10.3389/fimmu.2017.01868)
Supplement: Supplementary file 2 [file Image_2.PDF]

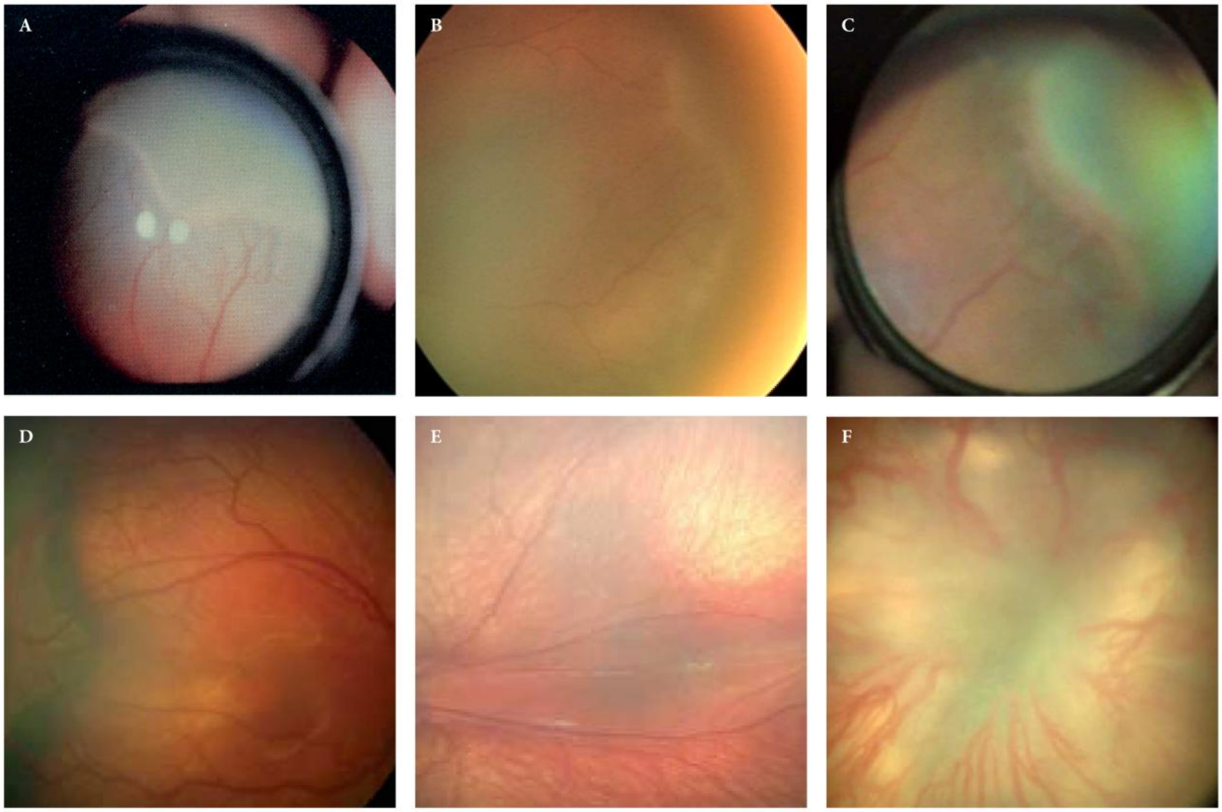

Supplementary figure 2: Fundus picture of ROP patients (screened in L V Prasad Eye Institute) showing different grades of severity of ROP: A) Stage 1:Demarcation line, B) Stage 2: Ridge between vascularized and vascularized retina, C) Stage 3: Extraretinal neovascularization, D) Stage 4a: Extra foveal partial retinal detachment, E) Stage 4b: Partial foveal retinal detachment, F) Stage 5: Total retinal detachment.
