# Supplementary material for: Abnormal Complement Activation and Inflammation in the Pathogenesis of Retinopathy of Prematurity
Source: Front Immunol. 2017 Dec 22;8:1868. doi: 10.3389/fimmu.2017.01868 (PMC5743907; doi:10.3389/fimmu.2017.01868)
Supplement: Supplementary file 3 [file Table_1.docx]

**Supplementary table 1. Demographics of the study participants in comparision with the previous studies**

|  | **Present study** | | **Study by Mohamed *et al*** | | **Study by Hernett *et al*** | |
| --- | --- | --- | --- | --- | --- | --- |
| **Study subjects** | ROP cases | Premature controls | ROP | No ROP | ROP | Premature controls |
| **Sample size** | 189 | 167 | 102 | 228 | 593 | 264 |
| **Male (n)** | 58.5%(110) | 52.7% (97) | 54.40% | 55.80% | 47.8% (284) | 43% (113) |
| **Female (n)** | 41.5% (78) | 47.3% (87) | 45.60% | 44.20% | 52.11% (309) | 57.1% (151) |
| **Mean gestational age (weeks ± SD)** | 29.9±2.01 | 31.9±2.1 | 26±2 | 28±2 | Prethreshold ROP=25.7± 1.7, Threshold ROP 24.5± 1.2 | 27.1±1.9 |
| **Mean birth weight (kg± SD)** | 1.28±0.28 | 1.46±0.36 | 0.884±0.337 | 1.192±0.389 | Prethreshold ROP= 0.758± 0.13, Threshold ROP 0.697± 0.125 | 0.823.6± 0.126 |
| **Severity of ROP** | Prethreshold ROP= 40, Threshold ROP= 148 | Mature retina | Prethreshold ROP=102,  Threshold ROP=0 | Mature retina | Prethreshold ROP=467,  Threshold ROP=126 | Mature Retina |
