# Supplementary material for: Abnormal Complement Activation and Inflammation in the Pathogenesis of Retinopathy of Prematurity
Source: Front Immunol. 2017 Dec 22;8:1868. doi: 10.3389/fimmu.2017.01868 (PMC5743907; doi:10.3389/fimmu.2017.01868)
Supplement: Supplementary file 4 [file Table_2.docx]

| **Gene** | **Chromosomal location** | **Number of SNPs analyzed** |
| --- | --- | --- |
| *CXCR4* | 2q21 | 4 |
| *AGTR1* | 3q21-25 | 16 |
| *EDN1* | 6p24-p23 | 9 |
| *EPO* | 7q21 | 6 |
| *ANGPT2* | 8p23.1 | 29 |
| *CETP* | 16q21 | 31 |
| *CXCL12* | 10q11.1 | 7 |
| *EPAS1* | 2p21-p16 | 26 |
| *HIF1A* | 14q21-q24 | 27 |
| *TSPAN12* | 7q31 | 30 |
| *VEGFA* | 6p12 | 25 |
| *IHH* | 2q33-q35 | 8 |
| *H2AFX* | 11q23.2-q23.3 | 6 |
| *GP1BA* | 17p13.2 | 8 |
| *PRELP* | 1q32 | 2 |
| *OPTC* | 1q32.1 | 16 |
| *MMP9* | 20q11.2-q13.1 | 22 |
| *C2* | 6p21.3 | 2 |
| *CFB* | 6p21.3 | 8 |
| *C3* | 19p13.3-p13.2 | 20 |
| *FBLN5* | 14q32.1 | 18 |
| *CFH* | 1q32 | 38 |
| *TGFB1* | 19q13.1 | 6 |
| *SERPINF1/ PEDF* | 17p13.1 | 8 |
| *MMP2* | 16q13-q21 | 11 |
| *TBX5* | 12q24.21 | 1 |

**Supplementary table 2. Showing the chromosomal location and number of SNP used in 26 targeted genes**
