# Supplementary material for: Abnormal Complement Activation and Inflammation in the Pathogenesis of Retinopathy of Prematurity
Source: Front Immunol. 2017 Dec 22;8:1868. doi: 10.3389/fimmu.2017.01868 (PMC5743907; doi:10.3389/fimmu.2017.01868)
Supplement: Supplementary file 5 [file Table_3.docx]

**Supplementary table 3: Primers used for RT-qPCR**

| **Genes** | **Forward primer (5' to 3')** | **Reverse primers (5' to 3')** |
| --- | --- | --- |
| *VEGF165* | ATCTTCAAGCCATCCTGTGTGC | CAAGGCCCACAGGGTTTT |
| *C3* | TCACCGTCAACCACAAGCTGCTACC | TTTCATAGTAGGCTCGGATCTTCCA |
| *Hif1-α* | CCAGCAGACTCAAATACAAGAACC | TGTATGTGGGTAGGAGATGGAGAT |
| *Β-actin* | CATGTACGTTGCTATCCAGGC | CTCCTTAATGTCAC GCACGAT |
| *IL1 beta* | AGCTGATGGCCCTAAACAGA | GGAGATTCGTAGCTGGATGC |
| *BAX* | TGCTTCAGGGTTTCATCCAG | GGCGGCAATCATCCTCTG |
